# Emotional exhaustion and workload predict clinician-rated and objective patient safety

**DOI:** 10.3389/fpsyg.2014.01573

**Published:** 2015-01-22

**Authors:** Annalena Welp, Laurenz L. Meier, Tanja Manser

**Affiliations:** ^1^Industrial Psychology and Human Factors, Department of Psychology, University of FribourgFribourg, Switzerland; ^2^Department of Psychology, University of FribourgFribourg, Switzerland; ^3^Institute of Patient Safety, University Hospital BonnBonn, Germany

**Keywords:** clinician burnout, patient safety, standardized mortality ratios, length of stay, intensive care unit

## Abstract

**Aims**: To investigate the role of clinician burnout, demographic, and organizational characteristics in predicting subjective and objective indicators of patient safety.

**Background**: Maintaining clinician health and ensuring safe patient care are important goals for hospitals. While these goals are not independent from each other, the interplay between clinician psychological health, demographic and organizational variables, and objective patient safety indicators is poorly understood. The present study addresses this gap.

**Method**: Participants were 1425 physicians and nurses working in intensive care. Regression analysis (multilevel) was used to investigate the effect of burnout as an indicator of psychological health, demographic (e.g., professional role and experience) and organizational (e.g., workload, predictability) characteristics on standardized mortality ratios, length of stay and clinician-rated patient safety.

**Results**: Clinician-rated patient safety was associated with burnout, trainee status, and professional role. Mortality was predicted by emotional exhaustion. Length of stay was predicted by workload. Contrary to our expectations, burnout did not predict length of stay, and workload and predictability did not predict standardized mortality ratios.

**Conclusion**: At least in the short-term, clinicians seem to be able to maintain safety despite high workload and low predictability. Nevertheless, burnout poses a safety risk. Subjectively, burnt-out clinicians rated safety lower, and objectively, units with high emotional exhaustion had higher standardized mortality ratios. In summary, our results indicate that clinician psychological health and patient safety could be managed simultaneously. Further research needs to establish causal relationships between these variables and support to the development of managerial guidelines to ensure clinicians’ psychological health and patients’ safety.

## INTRODUCTION

Safe patient care and care providers’ psychological health are central concerns of healthcare organizations. While past research shows that these two organizational outcomes are both at unsatisfactory levels (de Vries et al., 2008; Estryn-Behar et al., 2011; Aiken et al., 2012), the potential connections between them have been largely neglected when designing interventions to improve either outcome. A scientific understanding of linkages between clinicians’ psychological health and patient safety might provide healthcare leaders with an opportunity to manage these two important organizational goals synergistically – clinician health and patient safety.

The main aim of this paper is to broaden our understanding of the relationship between clinician burnout as an indicator of reduced psychological health, and patient safety. Burnt-out clinicians might be a patient safety threat because they lack the necessary resources to perform their jobs (Schaufeli et al., 1995). Thus, reducing clinician burnout might not only alleviate well-known individual and organizational effects (e.g., turnover intentions or sick leave; Toppinen-Tanner et al., 2005; Heinen et al., 2013) but might offer a means to influence patient safety. Existing studies examining relationships between clinician psychological health and patient safety rely largely on safety indicators such as clinicians’ overall safety ratings (Ramanujam et al., 2008). These safety ratings are influenced by clinicians’ subjective perceptions and may differ from more objective data sources collected in the course of patient care, such as standardized mortality ratios. In order to monitor and improve patient outcomes, however, we also need to understand the factors impacting on objectively measurable safety indicators. Therefore, this study includes both objective and subjective patient safety indicators.

A further aim of this study is to explore the role of clinician demographic (e.g., professional role) and organizational characteristics (e.g., workload) that might be related to patient safety. By identifying modifiable constellations of clinician demographic and organizational characteristics in combination with clinician burnout this paper addresses a current gap in work design interventions, which are aimed at increasing patient safety.

To address this gap, our goal is to answer three questions: does clinician burnout predict patient safety? What is the role of demographic and organizational characteristics in predicting patient safety? Is burnout a predictor of patient safety over and above demographic and organizational characteristics? We will first provide the relevant theoretical background and describe the current state of research on clinician burnout and patient safety. Based on these foundations, we developed hypotheses concerning the relationships between burnout and demographic and organizational characteristics, and patient safety.

### PATIENT SAFETY

Patient safety is an important indicator of hospital performance. While there is some debate concerning the exact number and degree of severity of safety-related events, the general problem of compromised patient safety is widely accepted. For instance, de Vries et al. (2008) concluded from their systematic review of eight studies covering 74 485 patient records that around 10% of hospitalized patients experience an adverse event, about half of which could have been prevented. They estimated that 7% of patients who are affected by adverse events suffer lasting damage and another 7% die.

Patient safety is decreased if so-called preventable adverse events occur – i.e., adverse events not inherent to the patient’s condition but resulting from the provision of care (de Vries et al., 2008). Preventable adverse events comprise not only events that cause temporary or permanent damage or even death, but also those that have the potential to do so. In a safe healthcare system, preventable adverse events are minimized, and, if they occur, recovery from them is maximized (Emanuel et al., 2008). Patient safety can thus be broadly defined as “the avoidance, prevention, and amelioration of adverse outcomes or injuries stemming from the process of healthcare” (Vincent, 2012, p. 4).

Due to the complexity of studying patient safety, many studies use subjective safety indicators. Using subjective patient safety indicators has advantages: clinicians are experts in their work domain and may therefore be best suited to detect and evaluate events endangering patient safety during care that might be difficult for outsiders to observe. However, there are often barriers to accurately recalling or reporting adverse events (Pfeiffer et al., 2010). Thus, subjective patient safety indicators may be biased. Clinicians may base safety ratings on their own performance, which may not be representative for the entire unit. Subjective safety ratings and error reporting may also be influenced by clinicians’ current mental or emotional states (Jones and Johnston, 2012). Clinicians may have trouble remembering the frequency of safety-related events, especially when the period they are asked about is protracted (West et al., 2009), or be unaware of them altogether. Finally, many studies use only self-report data to investigate the impact of subjectively perceived work characteristics on subjectively perceived patient safety, which can result in common method bias (Podsakoff et al., 2012). An alternative to subjective safety indicators is objective patient safety data.

Research investigating burnout and objective patient safety is scarce. One reason for the lack of studies might be that reliable objective patient safety data are often difficult to obtain. Observations require a lot of resources and preventable adverse events can be difficult to identify (does the observed incident constitute an adverse event?) or define (could the event have been avoided?). Adverse events can further be identified from patient record reviews or critical incident reporting systems, neither of which capture the true occurrence rate. Finally, relevant data may not be accessible for ethical reasons, or simply not be available.

However, healthcare organizations increasingly collect relevant patient safety indicators such as length of stay and standardized mortality ratios (e.g., Hoffer Gittell et al., 2000; Wheelan et al., 2003; Brewer, 2006; Davenport et al., 2007; Merlani et al., 2011; Aiken et al., 2014). Instead of focusing on preventable adverse events and therefore on process indicators, these data actually represent unfavorable patient outcomes – i.e., they can serve as primary indicators for patient safety issues so severe that preventable adverse events actually did result in a prolonged hospital stay or even death.

The present study investigates patient safety in intensive care units (ICUs). Patients in ICUs are particularly prone to preventable adverse events due to their critical condition requiring a higher number of complex care interventions (Rothschild et al., 2005; Moyen et al., 2008; Kane-Gill et al., 2010; Seynaeve et al., 2011) and relevant outcome data such as length of stay and standardized mortality ratios, are routinely collected. Combining them with subjective safety ratings of clinicians, this approach compensates for the advantages and disadvantages of subjective and objective patient safety indicators and allows for comparative analyses. In line with the above definitions, length of stay, standardized mortality ratios, and clinician-rated patient safety are global indicators of reduced patient safety in the sense that the occurrence was not followed by optimal recovery, and clinicians are aware of such incidents.

### BURNOUT

Within the context of clinician health, this study focuses on clinician burnout. Burnout is a core aspect of reduced work-related psychological health and represents a severe, chronic strain response of the individual to enduring stress at work (Maslach and Jackson, 1981; Maslach et al., 2001). Burnout as defined by Maslach and Jackson (1981) consists of three dimensions: emotional exhaustion, depersonalization, and decreased personal accomplishment. Emotional exhaustion is considered the core dimension of burnout (Maslach et al., 2001). Emotionally exhausted employees feel fatigued and unable to face the demands of their job or engage with people. Depersonalization refers to emotional and cognitive disengagement from one’s job and a distant, cynical attitude toward it. The third burnout dimension, reduced personal accomplishment, describes the feeling of not being able to make a meaningful contribution and overall reduced efficacy at work (Maslach and Jackson, 1981).

The conservation of resources (CORs) theory (Hobfoll, 1989, 2002) is often drawn upon to explain burnout development. According to COR, strain develops if an individual is threatened with loss of material or psychological resources, actually loses them, or an imbalance develops due to resource investment without the appropriate resource gain. Hobfoll (2002) argues that burnout develops particularly in this third case. As a consequence, individuals are hesitant to invest in their jobs, they develop negative affective states and negative attitudes toward their clients and are less vigilant. In turn, performance may suffer (Halbesleben and Rathert, 2008; Halbesleben et al., 2008).

While originally theorized to be limited to the human services professions, which require employees to invest a lot of emotional resources into their clients (Maslach and Jackson, 1981), it has been established that burnout can develop based on a multitude of stressors inherent to the work itself (e.g., time pressure, low control), social interactions (e.g., role conflict, poor working relationships with colleagues or supervisors) or individual characteristics (e.g., high neuroticism, external locus of control; Maslach et al., 2001).

Burnout is highly prevalent in healthcare workers. A European study found that, depending on the country, between 10 and 78% of registered nurses suffer from burnout (Aiken et al., 2012) and there is evidence that numbers are rising (Arigoni et al., 2010). This rise being attributed to nursing shortages caused by cost-cutting and demographic changes (Duvall and Andrews, 2010).

Healthcare staff in acute care settings such as ICUs seem to be highly susceptible to experiencing burnout, since many of the factors that have been associated with burnout are present in their work environment. A study on burnout in physicians found that 52% of emergency physicians, compared to 42% of physicians working on ward, were burnt-out (Estryn-Behar et al., 2011). A variety of work characteristics may contribute to the increased levels of burnout in these settings. For example, the number of patients in critical conditions requiring extensive care is higher than in other care settings (Moyen et al., 2008; Brinkman et al., 2013). This may exhaust clinicians’ resources. In addition, patients in ICUs are often unable to communicate effectively, yet may be more agitated than less acute patients, thus requiring clinicians to invest even more time and emotional resources.

### PATIENT SAFETY AND CLINICIAN BURNOUT

Evidence of a relationship between burnout and objective performance is scarce across organizational settings (Taris, 2006), and healthcare is no exception. Studies investigating relationships between clinicians’ psychological health and patient safety are mainly based on clinician-rated patient safety rather than objectively measured patient safety indicators. For example, West et al. (2009) found that burnout in medical trainees was associated with higher recall of medication errors 6 months later. Similarly, burnt-out nurses report more adverse events (Teng et al., 2010). Other studies investigated recollection of adverse events (Squires et al., 2010) or errors (Prins et al., 2009).

Since this previous research was focused on subjective patient safety, little is known about the effect of clinician burnout on objective patient outcomes, with two exceptions (Schaufeli et al., 1995; Cimiotti et al., 2012). Schaufeli et al. (1995) found no effect on standardized mortality ratios, but did find an unexpected negative effect on length of stay. So, the findings on the limited previous research are mixed. We expand on prior studies by utilizing a larger sample including both nurses and physicians, analyzing all three burnout dimensions separately, and in addition, investigating the effect of demographic and organizational characteristics.

We assume that due to an imbalance between resource investment and resource gain, burnt-out clinicians may lack the energy or motivation to effectively perform their duties and are thus less able to provide safe patient care. Unsafe care processes might translate into increased patient mortality and length of stay, and reduced overall patient safety as perceived by the clinicians.

Nahrgang et al. (2011) generally argue that mental and physical energy levels in burnt-out employees are such that safe work behaviors are lessened and so the likelihood of errors and work-related injuries is increased. An explanation of this relationship for the healthcare setting is offered by Halbesleben and Rathert (2008) and Halbesleben et al. (2008). The authors propose two mechanisms by which burnout may lead to reduced patient safety: first, because of resource depletion, clinicians may be less vigilant so their cognitive functioning suffers meaning preventable adverse events are more likely to happen. Second, as clinicians develop negative attitudes toward their patients, they can be reluctant to invest energy into observing or communicating with them, which may lead to loss of important information and reduce the quality of patient care, as perceived by clinicians and patients (Halbesleben and Rathert, 2008; Halbesleben et al., 2008).

We follow this line of reasoning and discuss these mechanisms separately for each burnout dimension. By definition, emotionally exhausted clinicians feel fatigued and unable to cope with the demands of their job. Emotional exhaustion could thus exert its negative effect on patient safety via a lack of physical and cognitive ability to perform one’s duties. To prevent further depletion of resources, emotionally exhausted clinicians may only execute tasks that are absolutely necessary (Halbesleben et al., 2008; Demerouti et al., 2014), neglecting safety behavior. Furthermore, cognitive processes such as executive functions, attention, and memory are impaired in burnt-out individuals (Deligkaris et al., 2014). As a result, exhausted clinicians may be less able to process the cognitive demands of highly technical and often rapidly changing ICU environment, pay less attention to details, such as small changes in patient status and are more likely to commit errors.

Depersonalization may function as a (dysfunctional) coping mechanism (Sonnentag, 2005) by which clinicians mentally detach from their work environment in response to a demanding work situation when other coping options, such as physically distancing oneself from or changing the demands, are unavailable. Some authors stress the motivational aspect of depersonalization, arguing that as a mechanism to maintain personal resources, the unwillingness to exert any more effort is the foundation of disengagement from the job (Taris, 2006; Demerouti et al., 2014). This disengagement comprises a depersonalized, dehumanizing attitude toward patients and a cynical attitude toward one’s job. Overall, reduced willingness to perform and lower commitment to the job may lead to negligence of duties, paying less attention to important details and thus higher rates of adverse events. For instance, being negligent about hand hygiene could lead to hospital-acquired infections, or committing a medication error could lead to serious drug side effects.

If clinicians are depleted of the resources necessary to perform their jobs, their sense of personal accomplishment – the belief that they can complete their tasks and make a meaningful contribution in their job – might decrease. Personal accomplishment is conceptually close to self-efficacy – i.e., the conviction that one has the capabilities to successfully accomplish a challenging task (Bandura, 1977). Self-efficacious individuals show higher performance because they are more persistent, exert more effort and view tasks as challenging rather than a threat (Stajkovic and Luthans, 1998). We assume that clinicians’ performance might suffer due to the belief that they are not capable of accomplishing work-related tasks. Clinicians might not invest the energy required to provide safe patient care, for instance, by neglecting hand hygiene or double-checks during medication preparation. They might also be less persistent when dealing with unexpected problems, for instance, irregularities in a patients’ condition, which might lead to decreased safety.

In summary, patients may be at a higher risk of suffering a preventable adverse event due to clinician burnout. A higher number of preventable adverse events is associated with more complications, which can lead to a prolonged hospital stay or, in very severe cases, death. The effect of burnout affecting patient safety via adverse events leading to increased mortality and length of stay would thus indicate a serious threat to patients.

#### Hypothesis 1

Burnout is associated with patient safety. Specifically,

(a) emotional exhaustion and depersonalization are negatively correlated with clinician-rated patient safety, and personal accomplishment is positively correlated with clinician-rated patient safety.
(b) Emotional exhaustion and depersonalization are positively correlated with standardized mortality ratios, and personal accomplishment is negatively correlated with standardized mortality ratios.
(c) Emotional exhaustion and depersonalization are positively correlated with length of stay, and personal accomplishment is negatively correlated with length of stay.

### DEMOGRAPHIC AND ORGANIZATIONAL CHARACTERISTICS

In addition to burnout, we included clinician demographic and organizational characteristics as predictors of patient safety. Demographic characteristics are individual attributes defining the role of a clinician within the ICU, such as his/her profession. Organizational characteristics are attributes of the work context, such as workload. Both demographic and organizational characteristics vary considerably across ICUs (Merlani et al., 2011; Kirwan et al., 2013). The effect of burnout on patient safety might be masked by them, or they may be independent predictors of patient safety. Including demographic and organizational characteristics can increase the practical applicability of research findings by pointing to additional opportunities for interventions (e.g., optimal team composition with regard to experience levels; Gibbs et al., 1991). Therefore, we will investigate the relationship of the demographic characteristics professional role (nurse vs. physician), professional experience, and professional status (trainee vs. non-trainee and clinical leader vs. non-leader), and the organizational characteristics workload, predictability, and team professional experience with patient safety.

Previous studies showed that safety perceptions differ depending on professional role, status, and professional experience (e.g., Vogus and Sutcliffe, 2007; Chang and Mark, 2009; Cimiotti et al., 2012). Findings regarding the direction of these associations are, however, mixed (Wilson et al., 2012). On the one hand, it has been reported that nurse leaders who spend less time at the bedside but have more experience in detecting safety threats report lower safety levels (Singer et al., 2009; Wilson et al., 2012). On the other hand, there is evidence that clinicians who spend more time on actual patient care tasks and are more exposed to safety-relevant situations tend to have lower safety perceptions than those who work in non-clinical areas (Singer et al., 2009). Since these studies only used subjective safety ratings, we do not know if these perceptions of patient safety correspond to objective safety indicators.

Based on prior findings, we expect clinician-rated patient safety to be lower in clinicians that spend more time at the bedside, specifically nurses (as opposed to physicians), trainees, and clinicians without leadership status. Nurses tend to spend more time on the unit, with the patient or involved in patient care, than physicians and might therefore be more sensitive to safety risks. Trainees might be overwhelmed and insecure about their abilities, which could lead to lower safety perceptions. Clinical leaders spend less time at the bedside and are thus less exposed to safety-threatening situations, which could be associated with more positive perceptions of safety.

We also expect standardized mortality ratios and length of stay to be higher on units with a higher percentage of trainees and lower percentages of clinical leaders. Trainees tend to commit more errors (West et al., 2006) and, if not supervised accordingly, might pose a safety threat. We do not have any assumptions regarding the impact of the ratio of nurses to physicians on standardized mortality ratios and length of stay so will only perform exploratory analyses of this effect.

Lastly, professional experience might relate positively to patient safety (Blegen et al., 2001) as it enables the individual to process and integrate novel information more quickly and to lead colleagues (Yun et al., 2005; Chang and Mark, 2009). The impact of high team professional experience might be even more pronounced, because the pooled competence of the entire team might be able to compensate for errors or lapses of less experienced team members.

In addition to the above characteristics, we will explore the effect of the organizational characteristics workload and predictability on standardized mortality ratios and length of stay. We define workload and predictability as work demands – i.e., physical, psychological, social, or organizational facets associated with clinician’s jobs which require effort (Karasek, 1979; Demerouti et al., 2001). In contrast to team professional experience, high workload and low predictability make acute care settings such as ICUs particularly demanding (Moyen et al., 2008; Estryn-Behar et al., 2011; Brinkman et al., 2013) and vulnerable to safety problems. High workload is thought to be detrimental to safety performance due to increased cognitive, emotional or physical load. For example, Baethge and Rigotti (2013) showed that perceived time pressure in clinicians predicted decreased subjective performance. Schubert et al. (2013) found that nurses who rationed the amount of nursing care due to overload, also perceived safety to be lower. Common indicators of workload in healthcare studies are nurse-patient-ratios or staffing adequacy (Coetzee et al., 2013). In the present study, we employed a quantitative approach to workload by calculating the number of patient care interventions executed by nurses such as medication or monitoring, relative to the number of patients, as an indicator of workload.

Low predictability is an additional risk factor for poor performance and low patient safety. For instance, self-reported interruptions predicted failure to remember intended actions and lower subjective performance (Baethge and Rigotti, 2013). Observational studies in operating theatres linked unforeseen complications (so-called non-routine events) with clinical performance (Burtscher et al., 2011). Low predictability requires clinicians to process a large amount of additional information in a short time and may force them to deviate from the routine path and change their behavior (Manser et al., 2009; Schraagen, 2011), thus increasing cognitive load which in turn can lead to both decreased performance and patient safety. We include the proportion of unplanned admissions as an objective indicator of low predictability.

#### Hypothesis 2

Demographic and organizational characteristics are associated with patient safety. Specifically,

(a) Trainee status, non-leadership status, being a nurse, low professional experience, high workload, and low predictability are negatively correlated with clinician-rated patient safety.
(b) Trainee status, non-leadership status, low professional experience, high workload, and low predictability are positively correlated with standardized mortality ratios.
(c) Trainee status, non-leadership status, low professional experience, high workload, and low predictability are positively correlated with length of stay.

## MATERIALS AND METHODS

### PARTICIPANTS AND PROCEDURES

Ethics approval for this study was granted from both the departmental and cantonal ethics committees (75, 2013-06-03; 024/13-CER-FR, 2013-24-06). We recruited medical and nursing staff working in ICUs in Switzerland. Participants were 1425 nurses and physicians in 54 ICU teams distributed across 48 hospitals. Of these participants, 1130 were nurses, 243 physicians, and 52 did not provide information on their professional background. The sample was predominantly female (*N* = 1027), 364 were men, and 34 did not provide this information. Age ranged from 19 to 63 years (*N* = 1401, *M* = 39.13, SD = 10.14), and professional experience from 0 to 43 years (*N* = 1386, *M* = 12.56, SD = 8.93).

Data on clinician burnout and clinician-rated patient safety were collected via an online self-report questionnaire over the period of 1 month. Data on workload, predictability, and objective patient safety were obtained during the same time period from a standardized dataset routinely collected by each ICU and then submitted to a central database at the Swiss Society for Intensive Care Medicine (SGI). Written consent to participate as a unit was obtained from ICU leaders, who also functioned as local study coordinators who forwarded the online questionnaire to their colleagues and were responsible for transmission of the patient care and unit data to the SGI. Individual clinicians were asked for their consent to participate, assured complete anonymity and confidential handling of their data upon opening the online questionnaire.

### MEASURES

#### Patient safety

Patient safety was assessed via clinician-rated patient safety, length of stay and standardized mortality ratios. Clinicians were asked to rate their perception of the unit’s safety level with one item (“please give your unit in this hospital an overall grade on patient safety”) from the Hospital Survey Of Patient Safety Culture (HSOPSC; Sorra and Nieva, 2003) translated to German, French, and Italian (Pfeiffer and Manser, 2010; Bagnasco et al., 2011; Occelli et al., 2013). Answers were provided on a five-point Likert scale (1 *= failing,* 5* = excellent*). While increased length of stay does not represent patient harm *per se*, it is widely used as an indicator of adverse events or complications that necessitate a longer ICU or hospital stay (Hoffer Gittell et al., 2000; Brewer, 2006; Merlani et al., 2011). Both crude and standardized mortality ratios are frequently used as indicators for quality of care processes and patient safety (Tourangeau et al., 2006). Crude mortality ratios indicate the percentage of deceased patients compared to all patients. Standardized mortality ratios are adjusted for patients’ risk of death by including several characteristics reflecting the severity of their condition (Le Gall et al., 1993; Wheelan et al., 2003). Thus, standardized mortality ratios are considered to be more reliable than crude mortality ratios (Tourangeau and Tu, 2003).

#### Burnout

Clinician burnout was assessed with the Maslach Burnout Inventory-Human Services (MBI-HSS; Maslach et al., 1996) in its appropriate translations to German, French, and Italian (Dion and Tessier, 1994; Büssing and Glaser, 1998; Pisanti et al., 2013). The MBI-HSS consists of the three dimensions *emotional exhaustion* (nine items, sample item “I feel mentally exhausted because of my work”), *depersonalization* (five items, sample item “I doubt the significance of my work”), and a positively formulated subscale called *personal accomplishment* (seven items, sample item “I deal very effectively with the problems at my work”). Responses were given on a seven-point Likert scale (1 = *never*, 7 = *always*). Cronbach’s alphas were 0.87, 0.63, and 0.71 for emotional exhaustion, depersonalization, and professional efficacy, respectively.

#### Demographic and organizational characteristics

Demographic characteristics professional role (nurse vs. physician), professional status (trainee vs. non-trainee and leader vs. non-leader), and professional experience were taken from the online survey data. Trainees comprised nurses and physicians undergoing advanced training to specialize in intensive care, and leadership status was defined as senior nurses and physicians leading the ICU. Team professional experience (in years), workload, and level of predictability served as organizational characteristics. We aggregated participant’s professional experience from the online survey to the unit level as an indicator of team professional experience, divided by the number of participants per unit. Nursing care interventions per patient relative to the number of patients, served as an indicator of workload. Nursing care interventions – also called nine equivalents of nursing manpower (NEMS) are patient care tasks executed by nurses such as monitoring, intravenous medication, ventilation, or dialysis. They are frequently used as an objective workload indicator both for practical and research purposes (Reis Miranda et al., 1997; Rothen et al., 1999; Carmona-Monge et al., 2013). Furthermore, we used the proportion of unplanned admissions (i.e., ratio of patients whose admission to ICU was not planned divided by all admissions during the data collection period) as an indicator of low predictability at the workplace. Data on workload and predictability were extracted from the central database of the SGI.

#### Control variables

Previous studies showed that clinicians’ ratings of burnout and safety differ between males and females: males tend to report lower burnout (Merlani et al., 2011; Myhren et al., 2013) and errors (Prins et al., 2009; Klein et al., 2010; Myhren et al., 2013). Thus, we controlled for the effects of gender. In addition, age was included as a control variable to explore the effect of professional experience independent from age.

### ANALYSES

Clinician-rated patient safety was measured at the individual level. To account for the nested data structure (i.e., individuals nested in teams), effects on clinician-rated patient safety were investigated using multilevel analyses with HLM 6 (Raudenbush et al., 2004). Age and gender were entered as control variables. Continuous predictors; emotional exhaustion, depersonalization, personal accomplishment, workload, predictability, and age were grand mean centered. Demographic characteristics; professional role, trainee status, and leadership status and control variable gender were dichotomous and thus dummy coded (0 = *nurses, non-trainees, non-leaders, females*; 1 = *physicians, trainees, leaders, males*). We used the restricted maximum-likelihood procedure in HLM for estimating the fixed and random parameters and robust standard errors for the significance tests (Hox, 2010).

In contrast to clinician-rated patient safety, mortality ratios and length of stay were measured on the unit-level, hence, no nested data structure exists and OLS regression analyses using SPSS were conducted. To predict the unit level outcomes of mortality and length of stay, individual-level predictors emotional exhaustion, depersonalization, personal accomplishment, professional experience, and age were aggregated at the unit level by calculating the unit mean. Gender, professional role, trainee status, and leadership status were aggregated by calculating the percentage of male participants, trainees, leaders, and physicians. Stepwise regressions were performed. In the first step, control variables age and gender were entered into the regression equation. In the second step, demographic characteristics professional role, trainee and leadership status, and organizational characteristics team professional experience, workload, predictability were added. Finally, emotional exhaustion, depersonalization, and personal accomplishment were entered into the equation.

Three units were deleted from the sample based on an outlier analysis following recommendations by Aguinis et al. (2013). The final sample for analyses at the unit level consisted of 54 ICUs.

## RESULTS

### DESCRIPTIVE STATISTICS AND CORRELATIONS

Mean, SD, and zero-order Pearson correlations among all variables at both individual and unit levels are presented in **Tables 1 and 2**, respectively.

**Table 1:** Mean, SD, and Pearson correlation among Variables at the Individual Level (*N* = 1391).

	*M*	SD	1	2	3	4	5	6	7	8	9
1 Gender	–	–									
2 Age	39.13	10.14	0.10*								
3 Emotional exhaustion	2.73	0.87	0.03	0.01							
4 Depersonalization	2.27	0.77	0.14**	-0.08*	0.50**						
5 Personal accomplishment	4.78	0.50	-0.06*	0.11*	-0.33**	-0.44**					
6 Clinician-rated patient safety	3.71	0.62	0.08*	0.00	-0.25**	-0.16**	0.18**				
7 Professional experience	12.56	8.93	0.004	0.76**	-0.01	-0.13**	0.10**	-0.03			
8 Professional role (nurse vs. physician)	–	–	0.35**	0.03	0.10	0.03	0.02	0.14**	-0.16**		
9 (non-) Trainee status	–	–	0.05*	-0.26**	0.10**	0.08**	-0.05	0.06**	-0.31**	0.32**	
10 (non-) Leadership status	–	–	0.18*	0.19**	-0.05*	-0.08**	0.11**	0.06*	0.15**	0.22**	-0.06*

*p < 0.05 (one-tailed test); **p < 0.01 (one-tailed test). Dichotomous variables gender, professional role, trainee status, and managerial status are dummy coded (0 = nurses, non-trainees, non-managers, females; 1 = physicians, trainees, clinical leaders, males).

**Table 2:** Mean, SD, and Pearson Correlation among Variables at the Unit Level (*N* = 54).

	*M*	SD	1	2	3	4	5	6	7	8	9	10	11	12	13
1 Gender	–	–													
2 Age	39.35	3.54	0.10*												
3 Emotional exhaustion	2.68	1.29	-0.24	-0.10											
4 Depersonalization	2.26	1.34	-0.13	-0.27*	0.46**										
5 Personal accomplishment	4.76	1.16	-0.16	0.26*	-0.15	-0.57**									
6 Clinician-rated patient safety	3.70	1.22	0.02	-0.07	-0.33**	-0.02	-0.12								
7 Standardized mortality ratios	0.18	1.07	-0.02	-0.31*	0.27*	0.01	-0.04	-0.26*							
8 Length of stay	2.61	1.34	0.18	0.18	0.12	0.07	-0.07	0.15	0.11						
9 Team professional experience	12.64	3.58	-0.01	0.90**	-0.05	-0.17	0.30*	-0.13	-0.36*	0.14					
10 Physicians	18.14	15.65	0.42**	0.01*	-0.29*	-0.20	-0.05	-0.17	-0.10	0.16	-0.14				
11 Trainee status	14.61	10.16	0.14	-0.31*	0.07	0.15	-0.07	.03	0.11	-0.05	-0.35*	0.55**			
12 Leadership status	14.67	10.11	0.24*	0.29*	-0.18	-0.34*	0.07	-0.01	-0.10	0.16	0.24*	0.51**	0.06		
13 Workload	3.33	4.11	0.23*	0.42**	0.06	0.05	0.07	0.10	-0.19	0.80**	0.38**	0.17	-0.09	0.21	
14 Predictability	0.72	0.17	0.65	-0.08	0.01	-0.25*	-0.03	0.08	-0.04	0.17	-0.07	0.07	-0.18	-0.08	0.05

*p < 0.05 (one-tailed test); **p < 0.01 (one-tailed test). Dichotomous variables gender, professional role, trainee status, and managerial status are aggregated to percentages of males, physicians, trainees, and clinical leaders.

### PREDICTORS OF CLINICIAN-RATED PATIENT SAFETY

In order to test whether burnout, demographic and organizational characteristics predicted clinician-rated patient safety, we conducted a multilevel model (see **Table 3**). With regard to control variables, results showed that males rated patient safety higher than females (*B* = 0.12, *t* = 3.00, *p* = 0.003), but age did not have an influence (*B* = -0.001, *t* = -0.69, *p* = 0.59). All burnout components predicted clinician-rated patient safety (*B*_EE_ = -0.13, *t* = -4.52, *p* < 0.001, *B*_DP_ = -0.07, *t* = -2.11, *p* = 0.04, *B*_PA_ = 0.16, *t* = 3.38, *p* = 0.002). With regard to clinician-rated patient safety, hypothesis 1a was confirmed. In line with our assumption, physicians rated patient safety higher than nurses; *B* = 0.15, *t* = 2.95, *p* = 0.004). Contrary to our expectations, trainees (*B* = 0.12, *t* = 2.41, *p* = 0.016) rated patient safety higher than non-trainees. Professional experience (*B* = 0.002, *t* = 0.72, *p* = 0.47), leadership status (*B* = 0.03, *t* = 0.64, *p* = 0.52), workload (*B* = 0.003, *t* = 0.52, *p* = 0.61), and predictability (*B* = -0.12, *t* = -0.92, *p* = 0.36) did not have an effect on clinician safety ratings (see **Table 3**). Except for professional role, hypothesis 2a was not confirmed.

**Table 3:** Multilevel random slopes model predicting clinician safety ratings on burnout and unit characteristics.

		Clinician-rated patient safety
**Predictors**
Level 1	Age	-0.00
	Gender	0.12**
	Professional role	0.15**
	Professional experience	0.002
	Trainee status	0.12*
	Leadership status	0.003
	Emotional exhaustion	-0.13***
	Depersonalization	-0.07*
	Personal accomplishment	0.16**
Level 2	Workload	0.003
	Predictability	0.12

### PREDICTORS OF STANDARDIZED MORTALITY RATIOS

Contrary to hypothesis 2b, none of the demographic (nurse vs. physician, leadership or trainee status) or unit characteristics (workload, predictability, and team professional experience) predicted standardized mortality ratios (β_percentage_ _physician_ = -0.19, *t* = -0.80, *p* = 0.43; β_percentage_ _trainees_ = 0.06, *t* = -0.28, *p* = 0.78; β_percentage_ _leaders_ = 0.03, *t* = -0.19, *p* = 0.85; β_workload_ = 0.12, *t* = -0.82, *p* = 0.42; β_predicatiblity_ = -0.10, *t* = -0.61, *p* = 0.55; β_team professional experience_ = -0.77, *t* = -1.99, *p* = 0.54). However, we suspect that team professional experience was not a significant predictor because of its high correlation with age (*r* = 0.90, *p* < 0.001). We repeated the regressions excluding age as a control variable, resulting in the expected association of team professional experience with standardized mortality ratios (β = -0.39, *t* = -2.30, *p* = 0.03). Of the three burnout dimensions, only emotional exhaustion predicted standardized mortality ratios (β_EE_ = 0.39, *t* = -2.23, *p* = 0.03; β_DP_ = -0.24, *t* = -1.24, *p* = 0.22; β_PA_ = -0.10, *t* = -0.06, *p* = 0.96; see **Table 4**). Hypothesis 1b was thus partially confirmed.

**Table 4:** Results of regression analyses of standardized mortality ratios, length of stay, on burnout and unit characteristics (*N* = 54).

	Standardized mortality ratios			Length of stay		
Step and variables	Step 1	Step 2	Step 3	Step 1	Step 2	Step 3
1 Age	-0.32*	0.39	0.43	0.21	0.18	0.24
Gender	-0.17	0.08	0.10	0.17	-0.03	-0.04
2 Professional role		-0.35	-0.19		-0.03	0.01
Trainee status		0.14	0.06		0.01	0.01
Leadership status		0.10	0.03		-0.003	-0.04
Team professional experience		-0.75	-0.77		-0.34	-0.15
Workload		-0.03	-0.10		0.86***	0.84***
Predictability		-0.05	-0.12		0.12	0.15
3 Emotional exhaustion			0.39*			0.01
Depersonalization			-0.24			0.14
Personal accomplishment			0.01			-0.02
Δ*R*^2^	0.10	0.10	0.10	0.08	0.61	0.02
Adjusted *R^2^*	0.07	0.05	0.10	0.04	0.63	0.63

*Standardized regression coefficients are reported for the respective regression steps.*

Step 1 including control variables age and gender, step 2 including organizational characteristics, and step 3 including respective burnout dimensions.

*p < 0.05 (two-tailed test); **p < 0.01 (two-tailed test); ***p < 0.001 (two-tailed test).

### PREDICTORS OF LENGTH OF STAY

In line with hypothesis 2c, workload (β = 0.86, *t* = 9.96, *p* = 0.00; see **Table 4**) was related to longer patient stays, however, none of the other demographic and organizational characteristics predicted length of stay (β_percentage_ _physicians_ = 0.01, *t* = 0.06, *p* = 0.96; β_percentage trainees_ = -0.004, *t* = -0.29, *p* = 0.77; β_percentage_ _leaders_ = 0.01, *t* = -0.09, *p* = 0.93; β_workload_ = 0.84, *t* = 8.54, *p* < 0.001; β_predictability_ = -0.15, *t* = -1.61, *p* = 0.11; β_team professional experience_ = -0.38, *t* = -1.59, *p* = 0.12). The relationship between workload and length of stay remained significant when the three burnout dimensions were entered into the regression equation (β = 0.85, *t* = 9.79, *p* = 0.00). Again, due to the large correlation between team professional experience and age (*r* = 0.90, *p* < 0.001), we repeated the regressions excluding age from the analyses, but team professional experience did not predict length of stay (β = -0.17, *t* = 1.58, *p* = 0.12). Overall, hypothesis 2c was partially confirmed. None of the burnout dimensions predicted length of stay (β_EE_ = 0.01, *t* = 1.32, *p* = 0.90; β_DP_ = 0.10, *t* = 0.86, *p* = 0.39; β_PA_ = -0.04, *t* = -0.46, *p* = 0.65). Hypothesis 1c was not supported.

## DISCUSSION

Our study investigated relationships between clinician burnout and patient safety while incorporating the effects of demographic and organizational characteristics. It expands on results of previous investigations by contributing several new findings: we included burnout, demographic, and organizational characteristics to investigate their combined impact on patient safety and established that overall, burnout was a stronger predictor of patient safety than demographic or organizational characteristics. More specifically, we established a positive relationship between emotional exhaustion and standardized mortality ratios as an objective patient safety indicator. In addition, workload and trainee status predicted patient safety. Lastly, in contrast to most studies in this field, we included the two main professional groups in intensive care, nurses and physicians, to gain a more comprehensive insight into the relationships between clinician burnout on patient safety.

### THE ROLE OF BURNOUT IN PREDICTING PATIENT SAFETY

Overall, we found evidence that burnout is associated with patient safety. Emotional exhaustion was the main predictor of standardized mortality ratios as well as of clinicians’ patient safety ratings. Emotional exhaustion is the core dimension of the burnout construct and relates to the feeling of being exhausted, depleted of energy, and not being able to complete one’s tasks. Therefore, it might impact on patient safety in two ways: firstly, continually feeling exhausted may lead to a decreased self-assessment of one’s performance and hence to lower subjective ratings of patient safety. Secondly, it might shape clinical performance via reduced vigilance or increased response times, which in turn, could lead to higher mortality ratios and thus to objectively decreased patient safety.

High levels of burnout might not just pose a problem for individual clinicians, but for the entire team. Previous research has established that burnout levels between individuals working in the same ICU are very similar and that burnout might carry over from one team member to another (Bakker et al., 2005). A single burnt-out individual on an ICU may not necessarily pose a safety risk as co-workers may be able to support burnt-out individuals. But if the majority of a team is burnt-out, errors may be more likely to go unnoticed or not be intercepted by colleagues, which might increase the likelihood for patient harm prolonging ICU stay or even contributing to death.

An alternative explanation for this relationship is that if high mortality ratios exist in a unit despite the high effort invested into caring for these critically ill patients, it may pose an increased risk for developing burnout. Future studies with a longitudinal design are required to test for causal effects.

Depersonalization did not predict objective patient safety indicators. There are several possible explanations for this finding. From a conceptual point of view, emotionally distancing oneself from one’s work to some degree might be an appropriate coping mechanism in this emotionally demanding work environment that does not necessarily decrease patient safety. From a methodological perspective, some items of the depersonalization scale refer to distanced interactions with conscious patients – yet many patients in ICUs have altered levels of consciousness or have difficulties communicating. Therefore, the depersonalization scale might not be entirely applicable to the ICU context.

Personal accomplishment was associated with clinicians’ patient safety ratings, but not with the objective safety indicators (i.e., length of stay and standardized mortality ratios). Personal accomplishment is the feeling of doing something worthwhile at work and having reached goals important to oneself. It is less about actual clinical competence and skills, which might explain why we did not find an association with objective outcomes such as length of stay and standardized mortality ratios. Moreover, clinicians providing the best possible care in critical care might feel that they have accomplished something worthwhile in their career despite high mortality ratios. Also, the fact that personal accomplishment was correlated with professional experience and occupying a leadership position could imply that clinicians might gain a feeling of personal accomplishment from other, more status-related sources rather than from actual patient care.

In contrast to standardized mortality ratios, clinician-rated safety was associated with all burnout dimensions. There are several potential explanations for this finding: firstly, burnout scores and patient safety as perceived by clinicians are both self-report data. Even though we asked clinicians to rate patient safety in their unit they might have focused on their own performance as the more salient information. Therefore, a (perceived) decrease in personal performance due to burnout might have had an immediate effect on their safety ratings. In addition, subjective safety ratings may have been negatively biased due to burnt-out employees’ generally decreased psychological health (Hakanen and Schaufeli, 2012).

Secondly, the link between burnout and patient safety *outcomes* such as mortality might not be as immediate. For example, errors caused by decreased performance in the *process* of patient care might be compensated for by colleagues; thus never resulting in negative *outcomes*.

Even though not all burnout dimensions predicted all patient safety indicators, our core finding remains that a relationship exists between emotional exhaustion and standardized mortality ratios. Thus, their interplay should be taken into consideration when aiming to improve either outcome.

### COMPARING RELATIONSHIPS OF BURNOUT AND UNIT CHARACTERISTICS WITH PATIENT SAFETY

Emotional exhaustion was a predictor of standardized mortality ratios, even when controlling for objective unit characteristics (i.e., workload, predictability). This finding has both positive and negative implications. Higher workload was associated with longer patient stays, but units with high workload and an unpredictable environment did not have more negative subjective safety perceptions or increased mortality ratios. Thus, with regard to these objective patient safety outcomes, clinicians seem to be able to cope with unfavorable working conditions. This does not exclude the possibility that workload or low predictability may have a negative impact in the healthcare environment *–* high workload or an unpredictable environment might still pose stressors for clinicians that contribute to or at least increase the likelihood of medical errors. It should be seen as alarming that the relationship between emotional exhaustion and standardized mortality *–* a very severe safety outcome *–* does play such a strong role and was not masked by other factors. This suggests that clinicians who feel overwhelmed and cannot cope with their work cannot care for their patients effectively and therefore, patients may have a higher risk of dying.

Contrary to our expectations, professional experience predicted neither of the safety outcomes. However, the relationship between team professional experience and standardized mortality ratios was close to significance. We believe that multicollinearity issues between team professional experience and age prevented this relationship from reaching full significance. When age was excluded from the analyses, team professional experience predicted standardized mortality ratios, and we believe that professional experience contributes to patient safety and should thus be considered in staffing decisions. Although experienced teams were associated with lower mortality, experienced clinicians did not rate safety on their units higher. On the contrary, trainees judged safety to be higher than clinicians who had completed their education. Trainees may not be able to judge safety as accurately as their experienced colleagues; this in itself might pose a threat to patient safety.

### LIMITATIONS

This study was cross-sectional, therefore, no inferences about causal relationships can be drawn. Also, selection bias may have influenced the results: units or individuals with high burnout levels may have declined to participate due to stressful working conditions. Compared to other European countries (Aiken et al., 2012), burnout in our sample was rather low. However, our results seem representative since Aiken et al. (2012) also showed that (clinician) burnout rates in Switzerland are amongst the lowest in Europe. Finally, working conditions in ICUs are very different from other healthcare settings. Thus, we do not know if our results are transferable. Currently, the kind of detailed, objective outcome data necessary for this research is mainly only collect within high-risk specializations in hospitals. Improved availability of reliable and valid outcome data for other care settings would allow similar analyses in other healthcare contexts to be conducted.

### PRACTICAL IMPLICATIONS

Our results provide input for managerial decisions concerning team composition and burnout prevention in intensive care. Emotional exhaustion was associated with mortality and clinician safety ratings. In addition, depersonalization, and personal accomplishment were related to clinician safety ratings. These findings illustrate the importance of burnout prevention to ensure patient safety and prevent negative effects for the organization. Burnt-out clinicians may not only be unable to maintain appropriate safety levels, but also further deplete their personal resources in an attempt to do so. This may have significant consequences in the long term, such as long sick leave absences (Toppinen-Tanner et al., 2005), turnover (Heinen et al., 2013) or early retirement (Sutinen et al., 2005; Hasselhorn et al., 2008).

Trainee status was predictive of clinician-rated safety, and there was a tendency of an association between team professional experience with standardized mortality ratios. To ensure appropriate levels of safety it seems important to have an appropriately high level of experience available on the unit at all times, or to encourage less experienced team members to seek the support they need to provide safe patient care, and help them to judge their safety performance accurately. It seems important to control workload in order to decrease complications that might result in longer hospital stays and incur higher costs.

### OUTLOOK

The Institute of Medicine defined six dimensions of quality healthcare (safe, effective, equitable, patient-centered, timely, and efficient; Kohn et al., 1999). The last two dimensions explicitly include clinician health as an essential aspect of healthcare quality. They state that high quality healthcare is timely, i.e., avoiding delays that are harmful to either patient or clinician, and that it is efficient, i.e., avoiding wasting material resources and ideas or energy of care providers. Our results lend support to the assumption that there is no trade-off between maintaining either patient safety or clinician psychological health, but that it is necessary and feasible to keep both at satisfactory levels in order to provide safe patient care. This finding carries great potential: the interdependence between clinician psychological health and patient safety might open up opportunities for managing both outcomes synergistically *–* i.e., by the same interventions.

In order to do so, we need an improved understanding of the factors impacting on objective safety indicators. Therefore, to clarify the causal relationships between burnout, demographic and organizational characteristics and patient safety, future research will require longitudinal and interventional studies. These studies should include subjective and objective process and outcome indicators of patient safety, short- and long-term stress and psychological health measures, and change of parameters possibly influencing both psychological health and safety.

So far, there seem to be two major scientific approaches to the clinician psychological health – patient safety relationship. Many studies assume that burnt-out employees perform poorly and thus might endanger patients (e.g., West et al., 2006; Halbesleben and Rathert, 2008). Others focus on safety-related events and argue that committing an error in the process of healthcare might effect clinician psychological health in the form of short-term emotional or physiological distress (Keijsers et al., 1995; Merlani et al., 2011; Jones and Johnston, 2012). For instance, Merlani et al. (2011) assumed that high crude mortality ratios were associated with higher burnout. If these events are severe or occur repeatedly, chronic strain or even symptoms similar to those of post-traumatic stress disorder might develop (Rassin et al., 2005). We believe that clinician psychological health and patient safety influence each other and evolve together. To our knowledge, there are no quantitative studies addressing this vicious cycle, and very few explore causal relationships (West et al., 2009). It is essential to not only include safety outcomes, but also process safety indicators, such as medication errors or infections, because these process errors committed by burnt-out individuals may have been compensated for by a colleague during the care process. So even if they did not result in drastic outcomes such as mortality, they might still have harmed the patient. Also, subjective ratings, for instance in the form of diary entries, can be valuable, as they can help identify safety risk moments. Other factors, such as teamwork might influence both clinician psychological health and safety, or compensate for the effects of burnout.

## CONCLUSION

To our knowledge, this is the first study that links clinician burnout with increased standardized mortality ratios and subjective patient safety indicators while incorporating demographic and objective organizational characteristics. We have shown that patient safety and clinician burnout are dependent on one another. Furthermore, we identified different predictors for the safety outcomes; standardized mortality ratios, length of stay, and clinician-rated safety. Evidence was found that mortality adjusted for severity of disease is higher on units with high emotional exhaustion. Our results led us to the conclusion that clinician psychological health and patient safety could and should be managed harmoniously.

Our study furthermore highlights the importance of combining the two major lines of research exploring the clinician psychological health – patient safety relationship. While one view assumes that decreased psychological health hinders safety, the other argues that safety-related events lead to short- or long-term reduced psychological health in clinicians. Integrating both views is necessary to explore the causal relationships between clinician psychological health and patient safety. This will lead to more specific insights into how to simultaneously improve and manage these two central hospital outcomes.

## Conflict of Interest Statement

The authors declare that the research was conducted in the absence of any commercial or financial relationships that could be construed as a potential conflict of interest.
